# Aalto Gear Fault datasets for deep-learning based diagnosis

**DOI:** 10.1016/j.dib.2024.111171

**Published:** 2024-12-02

**Authors:** Zacharias Dahl, Aleksanteri Hämäläinen, Aku Karhinen, Jesse Miettinen, Andre Böhme, Samuel Lillqvist, Sampo Haikonen, Raine Viitala

**Affiliations:** a Department of Mechanical Engineering, Aalto University, Espoo, Finland; b Kongsberg Maritime AS, Borgundveien 340, 6009 Ålesund, Norway

**Keywords:** Torsional vibration, Lateral vibration, Vibration dataset, Condition monitoring, Intelligent fault diagnosis, Deep learning

## Abstract

Accurate system health state prediction through deep learning requires extensive and varied data. However, real-world data scarcity poses a challenge for developing robust fault diagnosis models. This study introduces two extensive datasets, Aalto Shim Dataset and Aalto Gear Fault Dataset, collected under controlled laboratory conditions, aimed at advancing deep learning-based fault diagnosis. The datasets encompass a wide range of gear faults, including synthetic and realistic failure modes, replicated on a downsized azimuth thruster testbench equipped with multiple sensors. The data features various fault types and severities under different operating conditions. The comprehensive data collected, along with the methodologies for creating synthetic faults and replicating common gear failures, provide valuable resources for developing and testing intelligent fault diagnosis models, enhancing their generalization and robustness across diverse scenarios.

Specifications Table

**Table:** 

Subject	Mechanical Engineering
Specific subject area	Gear failure dataset for rotary machinery, specifically azimuthing thruster. Dataset consisting of vibrational data.
Type of data	Raw, Processed.
Data collection	The data has been collected using an 9:1 scaled azimuth thruster testbench with 11 sensors; 2 torque transducers, 4 accelerometers and 5 rotational encoders. 2 Bosch Rexroth motors used to drive and simulate load on the system. The sensors and motors where hooked up to National Instruments DAQs and LabVIEW software was used to control the whole system.
Data source location	Arotor Laboratory at Aalto University
Data accessibility	Repository name: Aalto shim and Gear failure datasets Data identification number: **16kHz:** DOI: 10.17632/6cc4ctxthy.1 **3012HZ:** DOI: 10.17632/fywnj597d8.2 **ASD:** DOI: 10.17632/fsjhhrw2y8.1 Direct URL to data: **16kHz:**https://data.mendeley.com/datasets/6cc4ctxthy/1 **3012Hz:**https://data.mendeley.com/datasets/fywnj597d8/2 **ASD:**https://data.mendeley.com/datasets/fsjhhrw2y8/1 Instructions for accessing data: Data is compressed using .RAR or .7z. To uncompress .RAR can be done using with ex. The WinRAR for windows or RAR for MAC. The .7z compression can be uncompressed using 7zip.
Related research article	None.

## 1 Value of the Data

• This dataset has been collected on a 9:1 replica test bench of a maritime azimuth thruster [1], which was built to simulate the complex dynamics of a real thruster. Hence, the collected dataset resembles data that could be gathered from an actual thruster, enabling research on mechanical behaviour.
• The dataset has been collected using several different sensors, listed in Table 1 with comparisons to other datasets in literature. Increased amount of measurement sensors promotes the development of robust intelligent fault diagnosis (IFD) models that can analyse and diagnose gearbox faults across different setups [2].

**Table 1:** Dataset comparison, between CWRU [6], Paderborn [7], Pronostia [8], MFPT [9], SEU [10], AGFD and ASD. AGFD and ASD are the datasets described in this paper. Repeated installations refer to disassembly and assembly of faulty components. As seen, the AGFD is the only dataset in this table with repeated installations of the faulty components. AGFD and ASD also include the largest rpm range and number of loads per rpm.

	CWRU	Paderb.	Pronos.	MFPT	SEU	AGFD	ASD
# of Bearing faults	9	26	17	17	0	0	0
# of Gear faults	0	0	0	0	4	8	9
rpm range	1730–1797	900- 1500	1500–1800	1500	1200–1800	250–1500	250–1500
# of load torques per rpm	1	1	1	1	1	3	3
# of accelerometers	2	1	2	1	7	4	4
# of encoders	0	0	0	0	0	5	5
# of torque transducers	0	1	0	0	1	2	2
Repeated Installations	No	No	No	No	No	Yes	No
• There is a lack of comprehensive datasets containing detailed information on gear faults. This dataset addresses this gap, with a number of labelled conditions, displayed in Table 1.
• This dataset covers a large variety of operation conditions, including multiple installations of fault affected gear connections. Which is a quality, often lacking in existing datasets. This variability can aid in training of intelligent fault diagnosis models, from a wide variety of different setups and scenarios, improving the generalisation and robustness of these models [3].
• Datasets that is particularly useful for model generalization and domain shift research, ensuring predictive maintenance models can perform well across different data from real-world situations, are scares. The dataset presented I this study offers a publicly available dataset for this type of research.
• The datasets have already been utilized in few previous studies, including [4,5], but have not yet been published.

## 2 Background

Azimuth thrusters are commonly used as the main propulsion solution on maritime vessels [11]. They feature either a single or double bevel gear stage that is exposed to extreme forces under severe weather conditions, including ice loads, which can lead to wear on the system [12]. To avoid unnecessary downtime, identifying potential failures and estimating the remaining useful lifetime is important. Deep learning (DL) based fault diagnosis could facilitate the task and is a research field that has received much attention lately [3]. However, one of the main issues with developing accurate DL models useful in real-world applications is them generalising badly across operation conditions and multiple instances of the same machine. This is often due to the lack of training data [3]. Existing data sets such as CWRU [6], Paderborn [7], Pronostia [8], MFPT [9] and SEU [10] have been a staple in the development of DL models used for condition monitoring. However, these datasets could include more data from varied operating conditions and measurements from different sensors. Additionally, most datasets focus on bearing faults, creating a lack of datasets for gear faults specifically. Aalto Gear Failure Dataset (AGFD) and Aalto Shim Dataset (ASD) address these gaps by including a large range of varying operating conditions, a large rpm range and number of load torques, and being a gear failure datasets. Table 1 presents a comparison of earlier datasets to AGFD and ASD.

## 3 Data Description

**Fig. 1:**
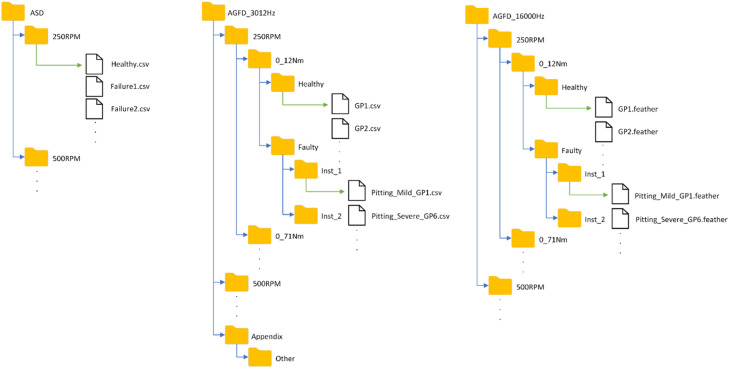
The folder structure of the datasets. Opened structure in Appendix folder. GP refers to gear pair.

The datasets are organised into the folder structure presented in Fig. 1 and compressed into a .RAR file to minimise storage requirements. The dataset is first split between ASD and AGFD, as ASD and AGFD are two separate datasets, measured at separate occasions. ASD introduces more artificial faults created by attaching shims to the gear. AGFD Is split into sampling frequency of 3012 Hz and 16000 Hz, introducing more realistic faults by replicating failure modes encountered in the industry. AGFD sampled at 16000 Hz is compressed into a .7z file. The fault modes will be covered in Section 4.3. The ASD folder structure is first split by driving motor speed, ranging from 250 to 1500 rpm. The specific details of driving motor speed and motor load are covered in section 4.2. The AGFD folder structures is first split by driving motor rotational speeds, and then by loading motor nominal torque, ranging from 0.12 to 1.31 Nm. Lastly, the dataset is split into Healthy and Faulty data folders. The Healthy folder directly holds the measurement files. The Faulty folder is further divided by installation number, shortened to Inst 1 and Inst 2. The measurements have been collected into .csv files, except for 16,000 HZ sampling frequency. The .feather file format was chosen for 16,000 Hz sampling frequency to save space, due to the size of the dataset. The naming of the files depends on if they belong to ASD or AGFD. ASD files are named after the class they represent, i.e., the healthy class is Healthy.csv, the first faulty class is Failure1.csv, etc. The AGFD files are additionally dependent on if it is a measurement of healthy or faulty condition. The healthy condition files are named after the gear pair used. The first gear pair that healthy data was collected for was designated gear pair 1 and the corresponding healthy measurement .csv files are named as GP1.csv. The faulty measurement files in AGFD file structure follow the same naming principle, but in addition to the gear pair the fault and severity are also included, i.e., mild pitting damage was replicated on gear pair 1, so the file name is Pitting_Mild_GP1.csv.

The measurements in the CSV files have been structured into 12 columns. An overview of the structure can be seen in Table 2. The first column contains the measurement time in seconds. Columns 2–6 contain encoder data, alternating between total angle turned in degrees. Each encoder also measured time on their own, but these were removed to decrees the size of each measurement file. The whole measurement time does match closely to the encoder measurement, with a small deviation. The encoders are numbered from 1 to 5 and their placements on the test rig is presented in Fig. 4. Columns 7–10 contain the measured accelerometer data. The placement of these sensors can be seen in Fig. 4. The last two rows, 11 and 12, contain the measurements from the torque transducers, labelled torq1 and torq2.

**Table 2:** The content and structure of measurement files.

Column no.	Column name	Content	Unit
1	Time	Timestamp	S
2	enc1_ang	Encoder 1 angle	Degree
3	enc2_ang	Encoder 2 angle	Degree
4	enc3_ang	Encoder 3 angle	Degree
5	enc4_ang	Encoder 4 angle	Degree
6	enc5_ang	Encoder 5 angle	Degree
7	acc1	Accelerometer 1	m/s^2^
8	acc2	Accelerometer 2	m/s^2^
9	acc3	Accelerometer 3	m/s^2^
10	acc4	Accelerometer 4	m/s^2^
11	torq1	Torque transducer 1	Nm
12	torq2	Torque transducer 2	Nm

A subfolder named Other can be found in the Appendix folder, in the AGFD 3012 Hz, which contains code used to analyse and process the data, as well as datasheets for sensors and motors. The Appendix folder also includes a figure of the full folder structure, showing all folders. A file named README.xlsx file is also included in the Appendix. This contains every run parameter, all the recorded anomalies for each measurement file that has been noted, which measurements are missing, as well as the file path of each measurement.

## 4 Experimental Design, Materials and Methods

The dataset was measured using the small scale maritime thruster test bench shown in Fig. 3. The dataset is divided into two different measurement sets: the first set, ASD, includes synthetic faults, with a total of 10 classes. The other set, AGFD, consists of replicated real bevel gear failure modes, and includes healthy and failure data, where also repeated installations have been done for each of the induced failure modes. This section will cover the small scale maritime thruster test bench that was used to collect the datasets, go over the process of the data acquisition, and explain the faults as well as how they have been replicated.

### 4.1 Thruster test rig setup

**Fig. 2:**
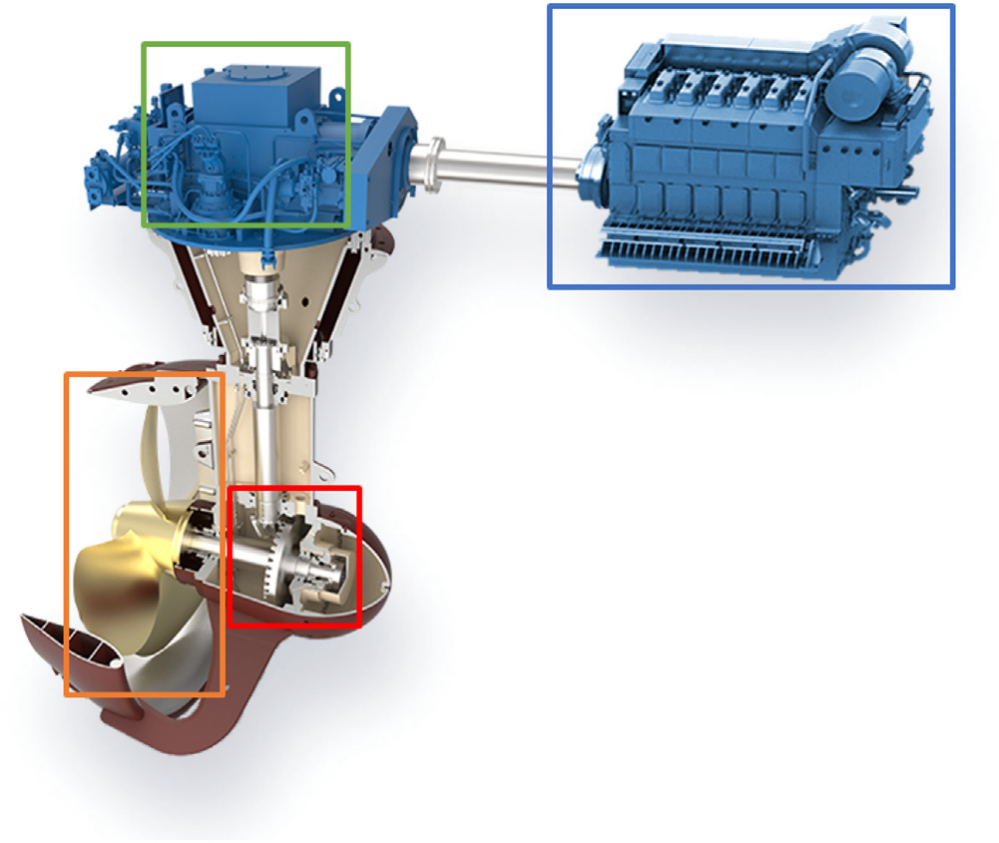
Azimuth z-drive thruster. The driving motor is marked in blue, the first gearbox in green, the second gear box in red and the propeller in orange. (For interpretation of the references to color in this figure legend, the reader is referred to the web version of this article.)

The small scale maritime thruster test bench, presented originally in [1], was designed to mimic the dynamics of its real life counterpart. This was achieved with the help of two servo motors connected with a drivetrain consisting of three gear boxes, couplings, shafts and appropriately massed flywheels. A block diagram of the drivetrain can be seen in Fig. 4 Bosch Rexroth MS2No6 synchronous servomotors were used as both the driving and load motors. Having a servomotor to simulate the propeller load made it easy to gather measurements from varying load conditions. A planetary gearbox, gearbox 3, with a 1:8 ratio was added to the load motor, so that propeller loads could simulate real operating conditions. The drivetrain was built to simulate a z-drive azimuthing thruster, meaning it has two 90-degree gearboxes between the two motors. An example of this kind of thruster can be seen in Fig. 2. The drive shaft has a flywheel added to it, which is connected using elastomer couplings. The drive shaft is then connected to the middle shaft, with a 90-degree gearbox, gearbox 1, that has a gear ratio of 3:1. The Middle shaft represents the vertical shaft, in-between gearbox 1 and 2 (see Figs. 2 & 4), of an azimuth thruster which is then connected to the propeller shaft with another 90-degree gearbox, gearbox 2, having a gear ratio of 4:1. Another flywheel is placed on the propeller shaft. Several bellow couplings has also been used between a number of components to reduce the effect that the motors have on the collected data of the sensors. In total, there are 11 sensors attached to the powertrain, which are presented in Table 3, the placements of the sensors are shown in Fig. 4. Fig. 5 shows healthy state torque measurement of one rotation of the propeller shaft using T1 sensor. For more details on thruster test rig setup please refer to [1].

**Fig. 3:**
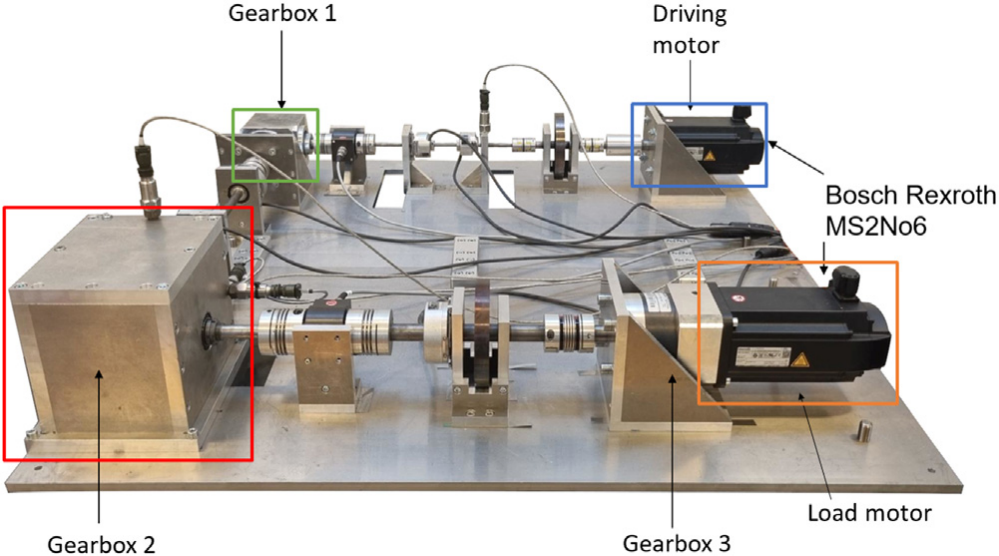
Small scale maritime thruster test bench. Corresponding components to a real azimuth thruster as seen in Fig. 2 and marked with the same colours. (For interpretation of the references to color in this figure legend, the reader is referred to the web version of this article.)

**Fig. 4:**
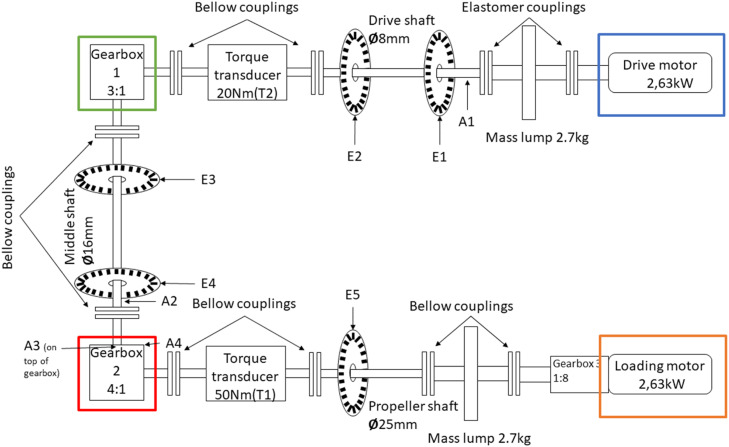
Downsized azimuth thruster model topology with sensors and components placement [1]. Corresponding components to a real azimuth thruster as seen in Fig. 2 and marked with the same colours. (For interpretation of the references to color in this figure legend, the reader is referred to the web version of this article.)

**Table 3:** Sensor types, naming, and designation.

Name	Type	Sensor
E1, E2, E3, E4, E5	Encoder	Heidenhain incremental ERN 120, ERN 420
T1, T2	Torque Transducer	Strain gauge-based ETH-Messtechinc
A1, A2, A3, A4	Accelerometer	Hansford HS-100 series

**Fig. 5:**
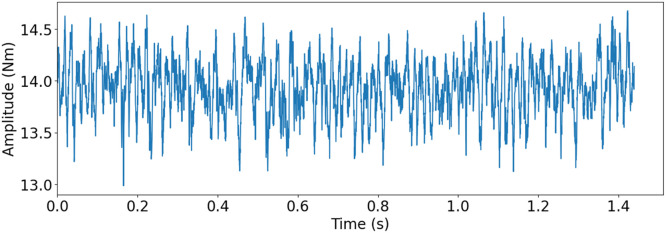
Torque measurement of one rotation of the propeller shaft with T1 torque sensor for healthy state. Drive motor speed at 500 rpm, load motor at 1.31 Nm and 3012 Hz sampling frequency.

Since the test bench is designed to simulate an azimuthing thruster, it was not desired to completely eliminate motor vibrations and other disturbances on the collected data. These factors introduce variability that appear during operation of a real-world thruster, making the dataset more representative. Such noise during training of machine learning models can enhance the generalisation performance, ensuring that the models are more suited to handle a wider range of real-world scenarios.

### 4.2 Data acquisition

**Fig. 6:**
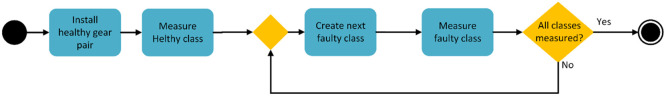
Measurement process flowchart for ASD.

**Fig. 7:**
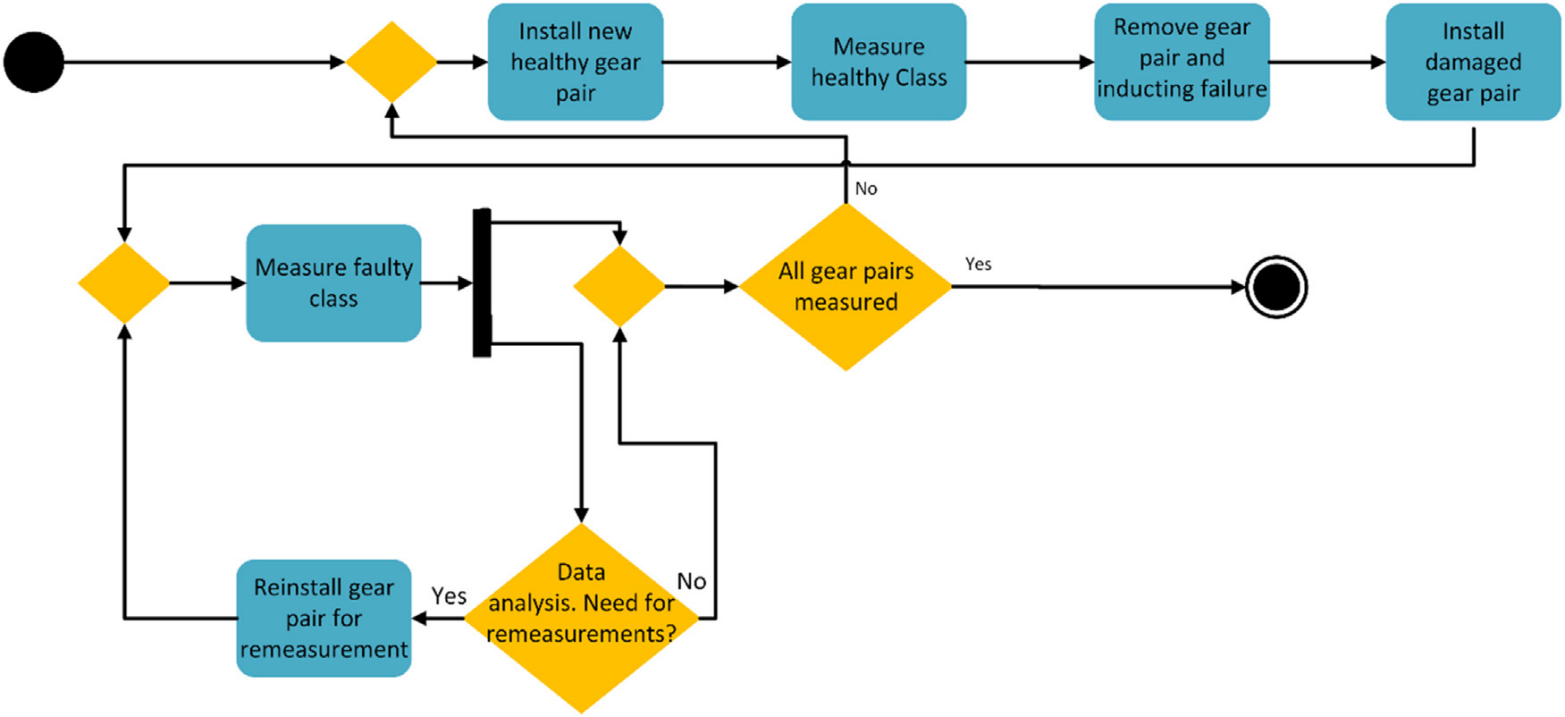
Measurement process flowchart for AGFD.

**Fig. 8:**
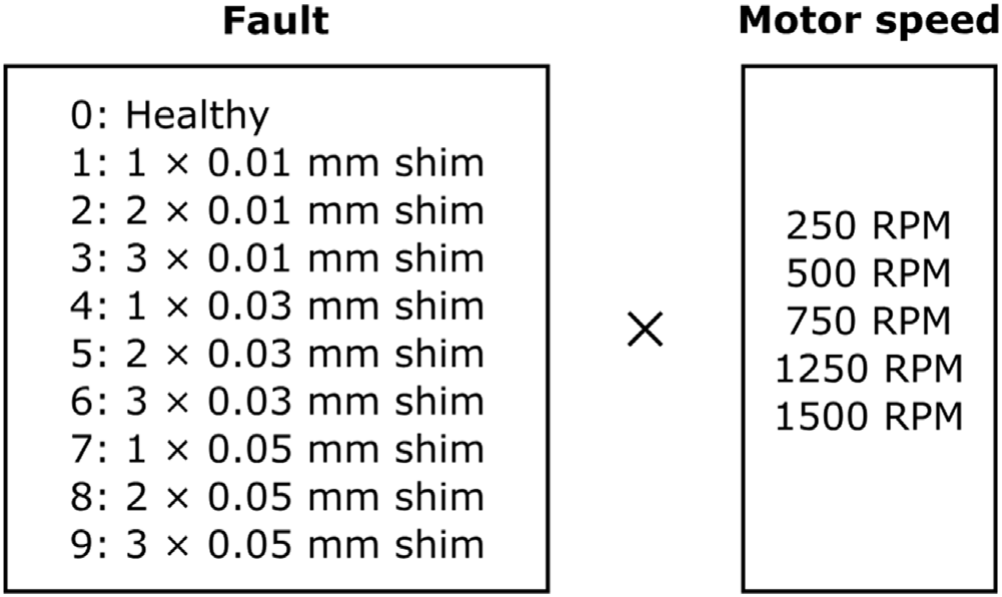
Parameters for the ASD dataset. The total number of classes is 10, consisting of each failure. Each class was run with 6 different motor speeds.

**Fig. 9:**
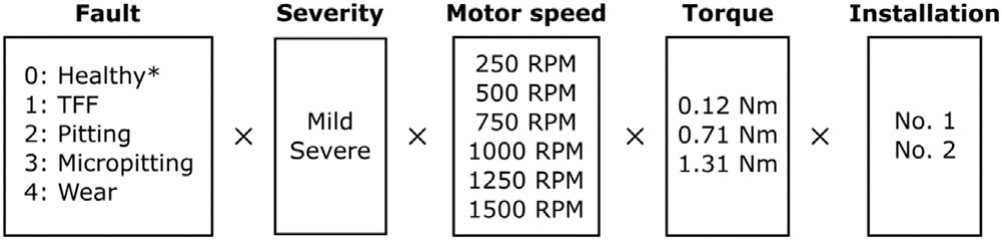
Parameters for the AGFD dataset. The total number of classes is 9, consisting of failure mode and severity. Each class was run with 6 different operation conditions, consisting of motor speed and torque. * The healthy class does not include a severity class.

Data acquired for deep learning purposes needs to follow certain criteria [2,3]. It is important to keep everything as similar as possible between class measurements to ensure that the model learns to differentiate classes by fault characteristics [2]. If the classes can be differentiated by some other learnable feature in the vibration, for example resulting from differences in the installation of the gears, results will be untrustworthy when used to rank how well different models can predict faults. Since multiple gears were installed several times and variation in the installations could cause unwanted noise, an installation instruction was developed and followed each time the bevel gears were assembled. As the aim of the repeated installations was to simulate the small differences in installation between real-world machines, some inaccuracies between installations were desired. This allows for model generalisation testing, as a DL model that works on one single machine and installation is not very useful. The process of measuring the ASD is presented in Fig. 6, and was done by first running the healthy state of the gear, with all the operating conditions. The operating conditions are presented in Fig. 8. A fault was then created on the pinion gear for the gear pair, and failure data was measured with the same operating conditions as for the healthy measurements, followed by the next failure condition. This process was continued until all failure conditions were measured. The length of the ASD measurements are 150 s. For acquiring the measurements for the AGFD, the structure presented in Fig. 7 was followed. To begin with, each pinion gear was paired with its own wheel gear and bearing. Before inducing faults in the pinion gears, a healthy measurement set was collected from each gear pair by installing them one by one and running them in the gearbox. Detailed parameters of these runs can be seen in Fig. 9. After obtaining measurements from the healthy gears, they were removed from the test bench. Subsequently, each pinion gear underwent the induction of failure modes, as described in Section 4.3. When the failure modes had been replicated, they were then installed into the gearbox again, by carefully following the aforementioned instructions. Failure data was collected, with the operating conditions shown in Fig. 9. Measurements were done first with sampling rates of 3012 Hz and then 16 kHz. For sampling rate 3012 Hz the healthy measurements are a total length of 110 s and the failure measurements are 230 s. For sampling rate 16 kHz the healthy measurements are a total length of 50 s and the failure measurements are 110 s. The shorter measurements for 16 kHz are due to restraints in data storage and was done to save space.

All data measurements were done by a testbench operator, that supervised the measuring process. During the measurements, the data were qualitatively inspected by plotting the sensor measurements in the time domain. The plots were inspected for large inconsistencies. Also, the ratios between encoders were calculated to make sure they stayed consistent. Maximum torque and vibration values were also checked to make sure they were on a reasonable level. If problems were noticed in a measurement, then a new run was done for the specific parameters used in the run. Software used for the analysis procedure was Matlab, and the code used can be found in the Appendix folder, in the folder labelled Other.

### 4.3 Faults and their production

The objective of this study was to replicate gear failure data that could be generated by a maritime thruster. In ASD, synthetic faults were first created by inserting shims between the gear contacts. Later, data from more realistic faults was desired. The realised AGFD replicated faults were replicated on the basis of the most typical gear faults found in the industry [[13], [14], [15]], the replications were qualitatively inspected to ensure their resemblance to real-life faults. Synthetic faults are presented in Fig. 8 and replicated faults in Fig. 9.

#### 4.3.1 ASD - synthetic faults

**Fig. 10:**
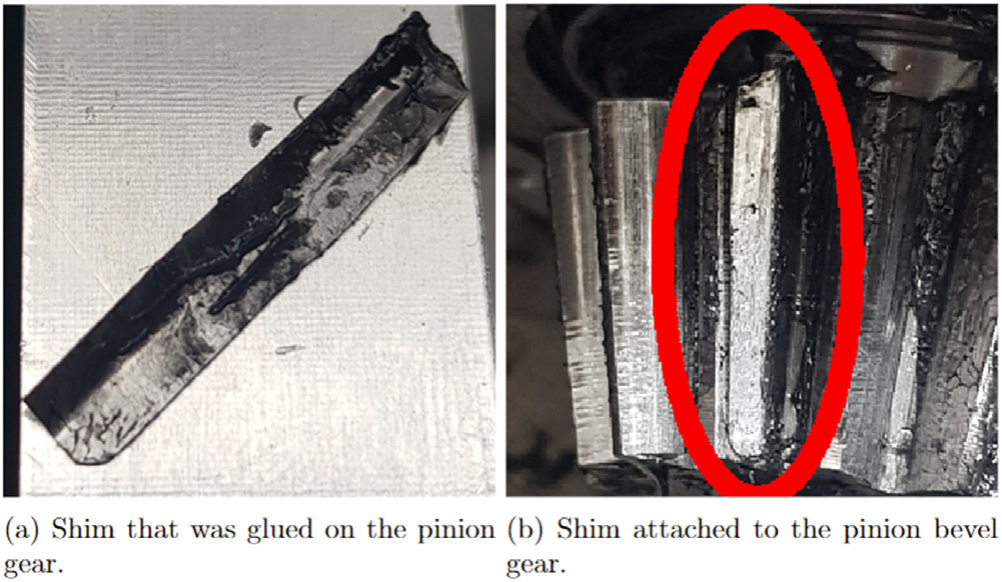
The creation of the synthetic faults.

**Fig. 11:**
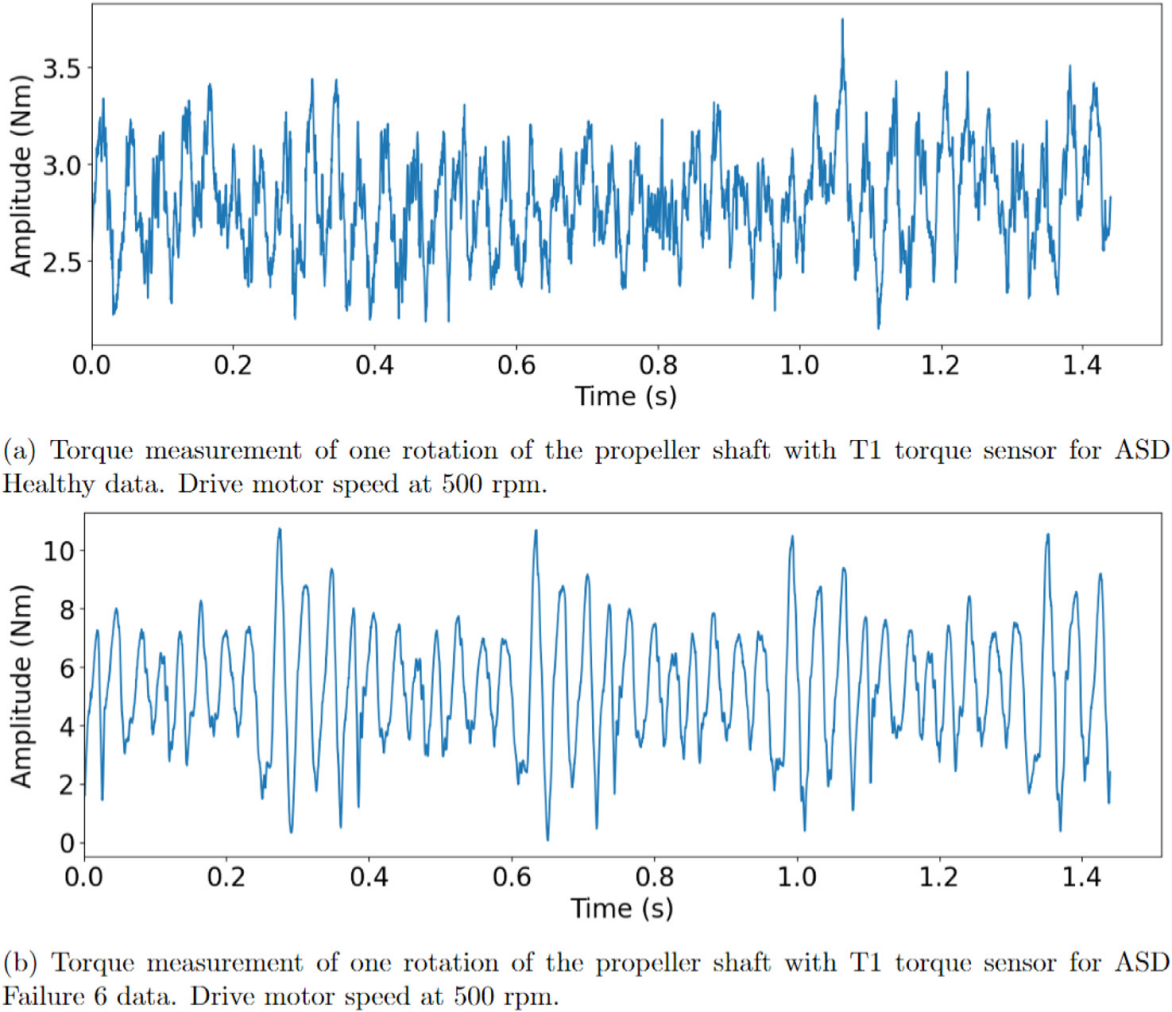
Rotation of propeller shaft for ASD dataset.

The synthetic faults were produced by adhesively gluing thin metal shims onto one of the teeth of the pinion gear. An example of a metal shim that was glued on can be seen in Fig. 10a and glued on to the pinion gear in Fig. 10b. A total of 10 different conditions were produced, the details of which can be seen in Fig. 8. The fault conditions were done by using 3 different thickness of shims, 0.01 mm, 0.03 mm and 0.05 mm, which all were varied between having 1–3 pieces. Hence, a total of 9 different fault conditions and one healthy condition was realised. Presented in Fig. 11a is one rotation of the propeller shaft, with ASD healthy data classification. Fig. 11b presents one rotation of the propeller shaft with ASD Fault 6 classification.

#### 4.3.2 AGFD – tooth flank fracture (TFF)

Tooth flank fracture is an extreme failure mode caused by fatigue and is the most frequent failure mode for bevel gears used in applications like azimuth thrusters [13]. The failure mode is caused by an increase in cyclic loading on a local area of the gear. It begins with microcracks forming during in-plane shear between the soft core and the hard case of case-hardened gear teeth underneath the load-carrying tooth flank. Then, the cracks propagate through the tooth in opening at a 40–50° angle towards the inactive flank's tooth root [14,15]. Most often, only one or very few teeth are affected. Eventually, under the acting shear stress, the crack will propagate to a stage where the tooth will fail to withstand the load on it, causing it to fracture as seen in Fig. 14a.

**Fig. 12:**
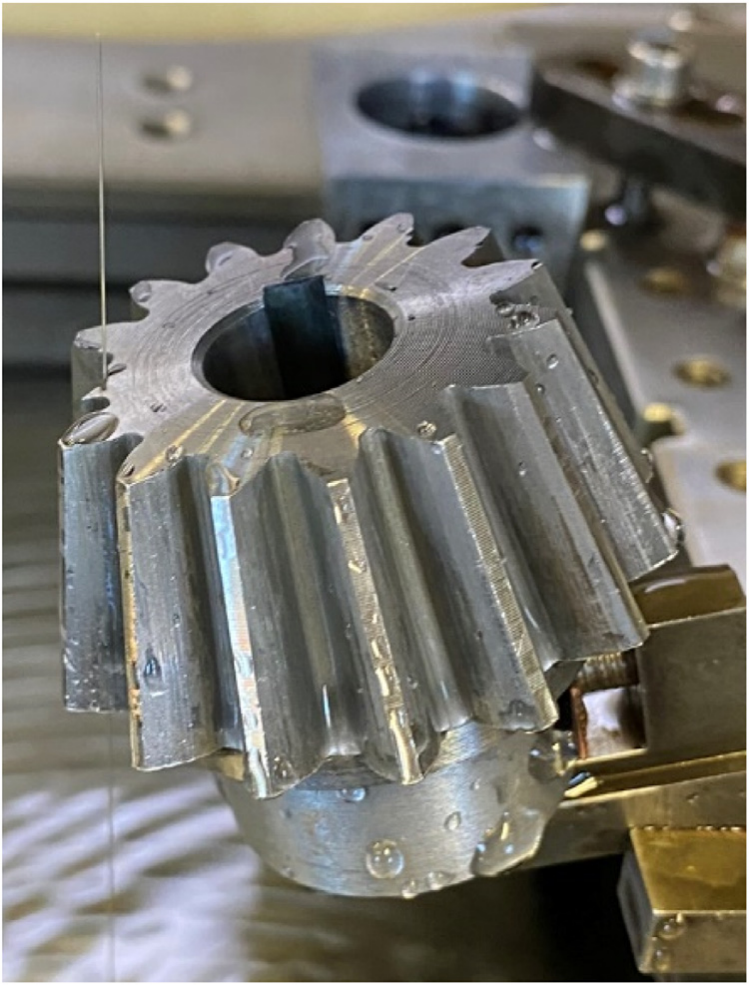
Replication of TFF with the help of an EDM.

**Fig. 13:**
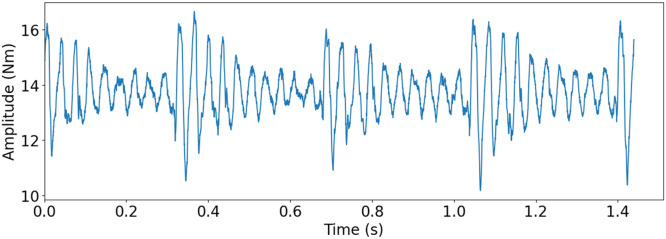
Torque measurement of one rotation of the propeller shaft with T1 torque sensor for severe TFF replication. Drive motor speed at 500 rpm, load motor at 1.31 Nm and 3012 hz sampling frequency.

To replicate the mild and severe tooth flank fracture, electrical discharge machining (EDM) was used (see Fig. 12). EDM cuts by generating sparks between the tool, an electrode, and the workpiece, being a completely contactless process. The EDM machine hence allows for small material removal, allowing for cuts that simulate the initial crack propagation and mild version of the tooth flank fracture damage. While thermal deformation would occur during this process, it was deemed to have only small to no effect on the result of the replication, due to how the replication where realised. For the mild version, a 5 mm incision was made from the active flank on the pinion gear at a 45-degree angle. The replication of the mild failure can be seen in Fig. 18a. For the severe version, a tooth was completely cut off from the pinion gear. The replicated severe failure can be seen in Fig. 18b. Fig. 13 shows torque measurement, for the replicated severe TFF failure, for one rotation of the propeller shaft measured with T1 torque sensor.

#### 4.3.3 AGFD - Pitting and spalling

**Fig. 14:**
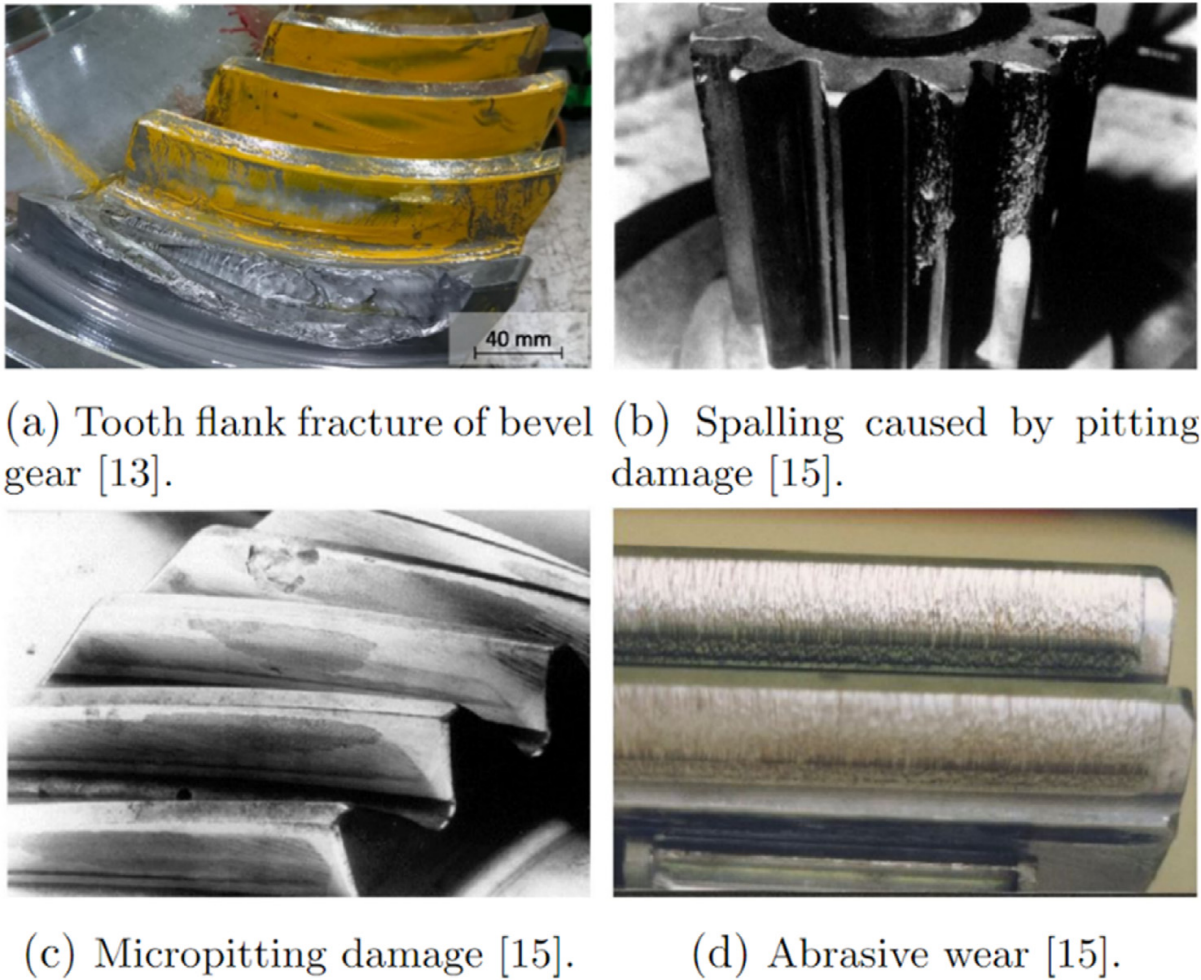
Depictions of real-world TFF, spalling caused by pitting, micropitting and abrasive wear.

**Fig. 15:**
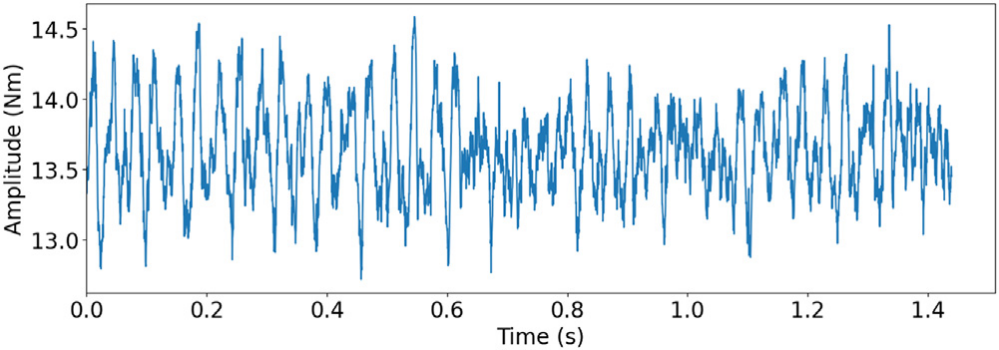
Torque measurement of one rotation of the propeller shaft with T1 torque sensor for severe pitting and spalling replication. Drive motor speed at 500 rpm, load motor at 1.31 Nm and 3012 Hz sampling frequency.

**Fig. 16:**
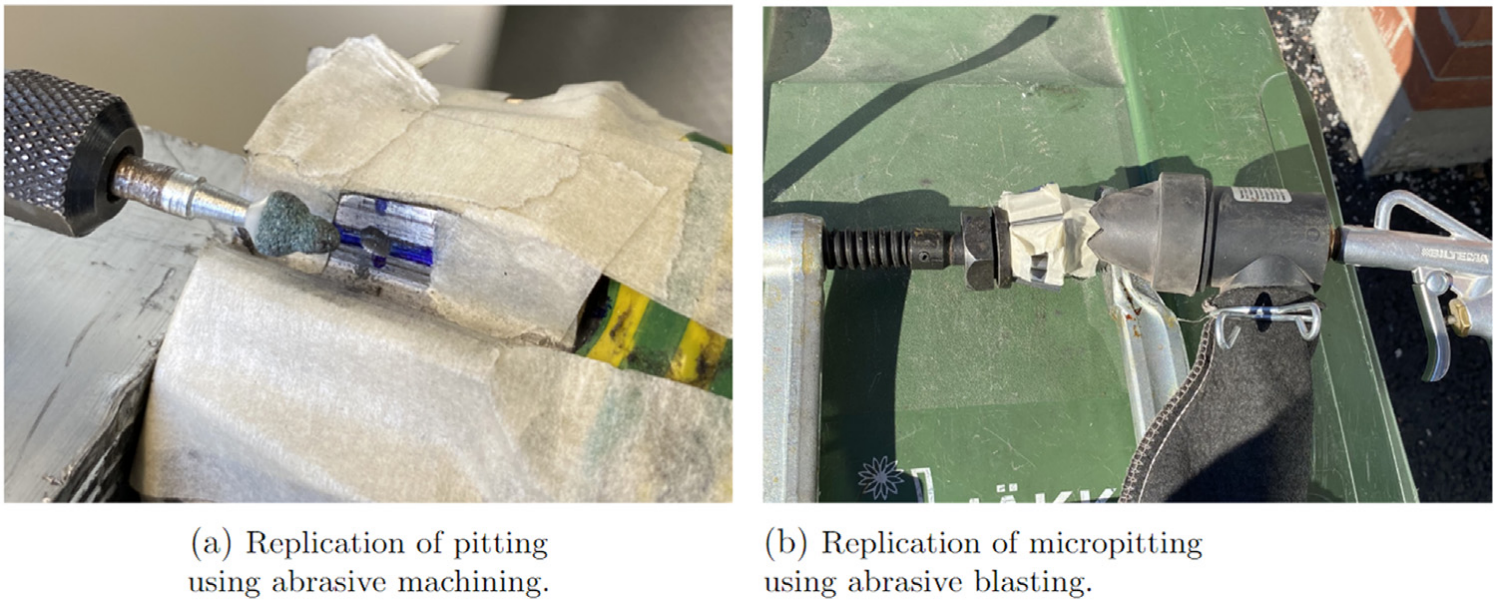
Replication of pitting and micropitting.

Pitting is a tooth contact fatigue type damage and a localized fault, typically only affecting a single or neighboring teeth in an azimuth thruster [16]. It is caused by Hertzian pressure repeatedly exceeding the material strength at the center of the pitch line, generating subsurface or surface cracks. Eventually, these cracks propagate into pit-shaped craters on the tooth flank just around the pitch line [[14], [15], [16]]. From there, the subsurface cracks propagate, creating an increasingly larger area of pitting. Eventually, spalling can occur and cause a greater amount of material to detach from the flank [15] (See Fig. 14b). Pitting, while not being the most critical failure mode for bevel gears in azimuthing thrusters, certainly is a prevalent failure mode. Pitting replication was done using abrasive machining with an electrical engraver. A spherical grinding stone was used to carefully grind the surface on one of the active flanks of the pinion gear, see Fig. 16a. For the mild version, a small pit was ground in the middle of the tooth at the pitch line height. Keeping the pit small was crucial to replicate the early stages of the failure. The replicated pitting can be seen in Fig. 18c. The severe version of the fault was created similarly but with a larger pit. In addition, material was also removed from this initial pit towards the addendum of the tooth, to replicate a spalling type of damage. The replicated spalling can be seen in Fig. 18d. Fig. 15 shows torque measurement, for the replicated severe pitting failure, for one rotation of the propeller shaft measured with T1 torque sensor.

#### 4.3.4 AGFD-micropitting

Micropitting can be identified on gears as a matte or frosted area of the tooth, typically in the dedendum of the pinion gear tooth, since it is the only region that experiences negative sliding [17]. In a mild case it often only affects one tooth, but when reaching a more severe state it affects the adjacent teeth. Micropitting is a surface failure mode, often caused by a failure in the lubrication between the gears [16]. However, micropitting also shares characteristics with regular pitting, in the sense that it is a local failure caused by stress peaks on the tooth flank [16]. The increase in stress often causes microcracks, which in themselves are not problematic, but with every rotation the negative sliding and the orientation of the cracks causes the oil to get trapped and pressurized, resulting in destructive material removal [17]. Micropitting is not regarded as a severe failure but is often the cause for other failures, and thus covered in this study.

**Fig. 17:**
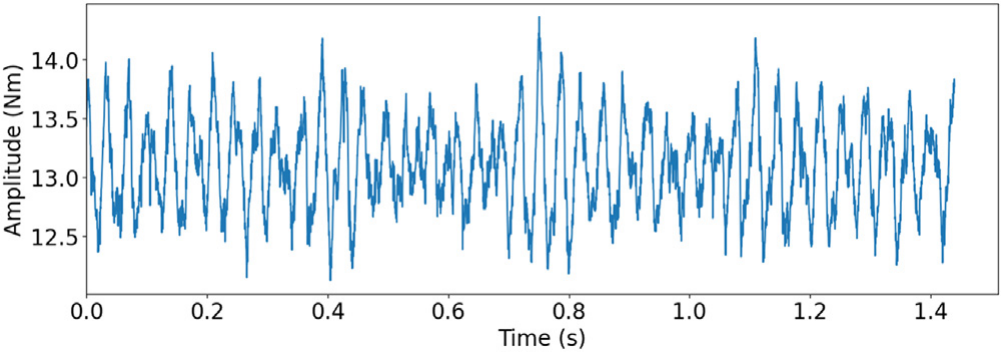
Torque measurement of one rotation of the propeller shaft with T1 torque sensor for severe micropitting replication. Drive motor speed at 500 rpm, load motor at 1.31 Nm and 3012 Hz sampling frequency.

**Fig. 18:**
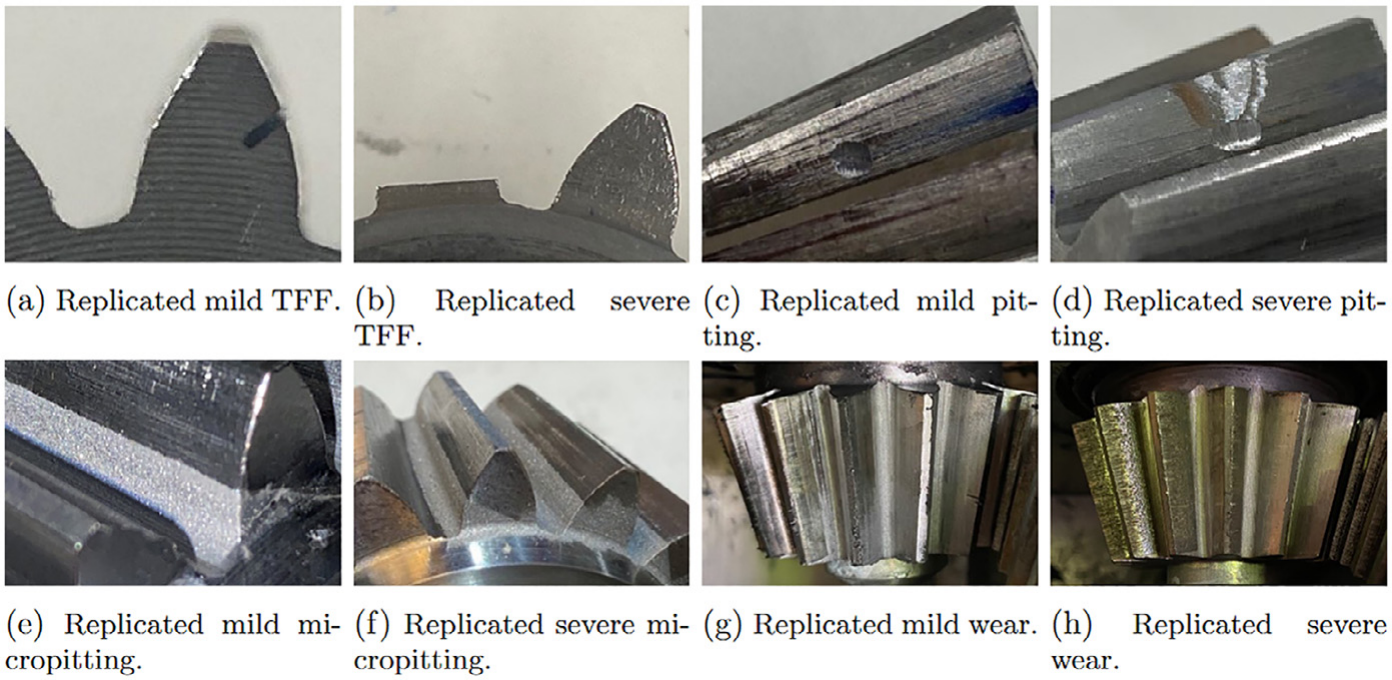
Replicated failures.

Since micropitting requires such a small amount of material removal, abrasive blasting was used to replicate the failure. The media used for blasting was sand, and air pressure was used to accelerate the media. The replication can be seen in Fig. 16b. The mild version of the fault replication had one tooth of the pinion gear blasted for 15 s. The severe version had three neighbouring teeth blasted for 60 s each. The replicated mild micropitting can be seen in Fig. 18e and sever micropitting can be seen in Fig. 18f. Fig. 17 shows torque measurement, for the replicated severe micropitting failure, for one rotation of the propeller shaft measured with T1 torque sensor.

#### 4.3.5 AGFD-wear

Wear in itself is not a single failure mode, but a collection of a variety of different surface failures [16]. In general, wear failures can often be identified as different kinds of score marks, depending on the wear type, affecting the surface of all teeth. A wear failure is caused by a combination of a failing oil film with the possibility of accompanying particles, like debris, in the oil [15,16]. The type of wear that occurs is often dependent on the operating conditions. Some examples of different kinds of wear that can occur are abrasive wear (see Fig. 14d), adhesive wear, polishing, corrosion, fretting corrosion, scaling, cavitation, erosion, and electrical discharge [15]. Sometimes, scuffing is also considered a wear failure [18]. For the purposes of this study, it is irrelevant whether scuffing is considered to be wear or not, as it has negligible difference to other wear failure modes when considering the expected gear excitations. The scuffing process is initialized from a failing oil film, which leads to an increase in friction upon contact between the pinion and wheel gear [16]. This increase in friction leads to micro-welding occurring between the teeth in contact.

The weld is then torn apart when the teeth disengage, resulting in destructive material removal [16]. Abrasive wear differs from adhesive wear in the sense that the leading factor for its cause is the presence of redundant particles [15,18]. The marks of abrasive wear are considerably similar to adhesive wear, being scrape marks in the relative motion direction. The difference is that abrasive wear marks are more significant, as they are caused by a sort of grinding effect [15,18]. Abrasive wear can also be identified by the deformation of the teeth, as the greater marks often push material over the edge.

**Fig. 19:**
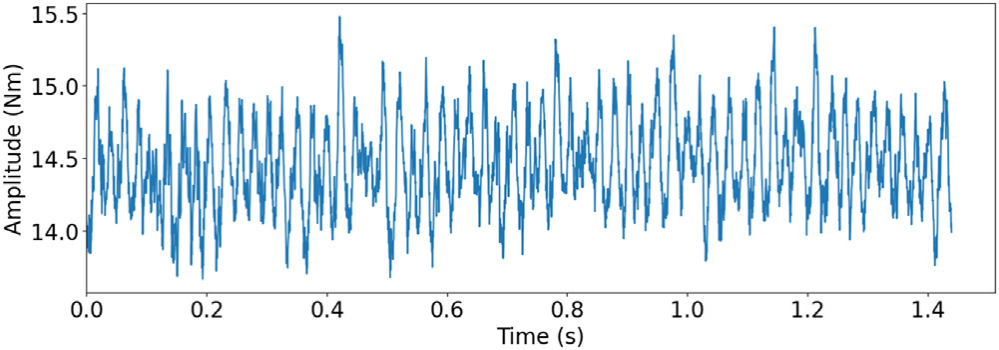
Torque measurement of one rotation of the propeller shaft with T1 torque sensor for severe wear replication. Drive motor speed at 500 rpm, load motor at 1.31 Nm and 3012 Hz sampling frequency.

Wear replication was done by simply having the gears assembled into the gearbox without proper lubrication, and then run at different speeds and loads for certain durations. The mild version was run for about 90 min, not allowing for a large amount of material removal. The severe version was run for about 430 min. For the mild version, marks were starting to appear on all teeth of the pinion gear, as seen in Fig. 18g, while the severe version had all the teeth completely covered in a lot of marks and is shown in Fig. 18h. The achieved wear was mostly due to the debris that was created and trapped in the gear contact. Qualitatively analysed, even though the failure created was abrasive wear, the measurements taken from the different sensors would be similar to signals generated by scuffing or adhesive wear. Fig. 19 shows torque measurement, for the replicated severe wear failure, for one rotation of the propeller shaft measured with T1 torque sensor.

## Limitations

The foremost limitation to ASD is that the classes do not accurately resemble real-life failures. One limiting aspect is that there are differences in the measured data between different gear pairs. This is most likely due to some error in the installation process, leading to two different installations having a different mean amplitude in the same operating conditions.

Another limitation is that some replicated faults in AGFD are not completely accurate to their real-life counterparts. This mostly concerns the severe TFF and micropitting failures. In reality, even though most of the tooth is removed when TFF occurs, the cut made should have been at a 40–50° angle. The replicated failure resembles more how a tooth interior fatigue fracture (TIFF) would appear [19]. However, the replicated fault still greatly represents the behaviour of an actual fault. Also, since severe micropitting in real-world applications can have material removal of up to 20 µm [15], using sandblasting might not have left enough of an imprint on the teeth. While ensuring similarity between the measured failure data and actual fault behaviour is difficult, the resulting replicated faults and data are qualitatively good.

Certain runs for the AGFD are missing and the measurement at 16 kHz sampling rate have some jumps in the measured data. More information is provided in the README.txt.

## Ethics Statement

The authors of this paper have read and followed the ethical requirements for publication in Data in Brief and ensures no human subject, animal experiments or any data collected from social media platforms has been used.

## Credit Author Statement

Zacharias Dahl*: Writing - Original Draft, Conceptualization, Methodology, Visualization and Data Curation, **Aleksanteri Hämäläinen:** Writing - Review & Editing, Conceptualization and Visualization, **Aku Karhinen:** Writing - Review & Editing and Conceptualization, **Jesse Miettinen:** Writing - Review & Editing and Conceptualization, **Andre Böhme:** Writing - Review & Editing and Conceptualization, **Samuel Lillqvist:** Methodology, **Sampo Haikonen:** Writing - Review & Editing and Methodology, **Raine Viitala:** Writing - Review & Editing and Supervision.

## Data Availability

Mendeley DataAalto Shim Dataset (Original data). Mendeley DataAalto Gear Failure Dataset 3012Hz (Original data). Mendeley DataAalto Gear failure dataset 16kHz (Original data).

## Appendix Supplementary materials

Image, application 1
